# From gene to plate in Indonesia: a food system framework for the triple burden of malnutrition

**DOI:** 10.3389/fpubh.2026.1755411

**Published:** 2026-03-12

**Authors:** Lestari Octavia, Intan Ria Nirmala

**Affiliations:** 1 Department of Information Systems, Universitas Gunadarma, Depok, Jawa Barat, Indonesia; 2 Department of Nutrition, Health Polytechnic of Kendari, Ministry of Health, Kendari, Indonesia

**Keywords:** food_system, framework, gene, malnutrition, plate

## Abstract

Global malnutrition has expanded to multiple causes, characterized by the coexistence of malnutrition problems. In many countries, child stunting/wasting occurs alongside rising obesity and diet-related non-communicable diseases in adults. The depletion of soil and crop minerals exacerbates hidden hunger worldwide. To address this problem, we need to invest substantial effort and adopt a broad perspective to transform the gene-to-plate continuum into a framework for a sustainable food system. This guidance aims to effect a fundamental shift in how the problem is conceptualized, moving beyond single-sector interventions to ensure year-round access to diverse, nutrient-dense foods through climate-smart agriculture, biofortification, and the use of local biodiversity. This conceptual paper develops the Gene-to-Plate framework, using Indonesia as the primary setting and comparative illustrations from Peru and South Africa, to propose and guide multisectoral policy in addressing malnutrition. Effective strategies involve community-based food programs, multisectoral policy integration, and strong nutrition networks. Case studies from Peru, Indonesia, and South Africa demonstrate that the development of local food systems and enhanced dietary diversity can sustainably mitigate malnutrition and decrease dependence on imports. Sustainable, locally grounded food systems are vital to achieving Sustainable Development Goals (SDGs) 2 and 3. The transformation from gene to plate—integrating production, distribution, and nutrition education—offers a long-term pathway to reducing malnutrition.

## Introduction

Malnutrition has emerged as a deeply entrenched global health crisis that transcends the conventional boundaries of hunger and food scarcity (1). Modern frameworks now identify it as a “triple burden,” which includes not only undernutrition—manifested by stunting, wasting, and underweight—but also a significant increase in overweight, obesity, and a range of micronutrient deficiencies frequently referred to as “hidden hunger” (2). The simultaneous presence of these conditions, often within the same country, household, or individual, signifies an era of swift demographic and dietary transformation (3). Many things make malnutrition worse and more complicated (4). The rapid growth of cities in low- and middle-income countries has transformed food environments, leading to increases in calorie consumption (5). People are now eating more energy-dense, nutrient-poor foods such as sugar-sweetened drinks, packaged snacks, refined grains, and fast food, rather than relying primarily on minimally processed foods or vegetables (6). These foods have too many calories and not enough important micronutrients, which makes adults overweight, obese, and sick with diet-related diseases. Many kids in the same places still eat diets that aren’t very nutrient-dense or varied (7). These nutrient-poor foods contribute to adult obesity and chronic disease, while leaving some children undernourished (8). At the same time, climate change and environmental degradation reduce agricultural productivity and exacerbate food insecurity (8–10). Economic shocks, such as unstable food prices, make it harder for people to eat healthy, especially for families that are already struggling (11). Social conflict further constrains the ability of communities to maintain regular food access and adequate nutrition.

Indonesia serves as the primary empirical context for this analysis; this study uses Peru and South Africa as comparative cases to demonstrate that analogous Gene-to-Plate dynamics manifest across diverse socio-political environments. The framework is designed as a transferable instrument that can be adapted for use in other nations facing the triple challenge of malnutrition and rapid transformation of food systems. Indonesia, Peru, and South Africa stand as stark examples of this multifaceted crisis. Despite notable improvements in food production and poverty alleviation, the nation continues to report high rates of stunted and wasted children, even as adult obesity and diet-related non-communicable diseases escalate (12). Intensive farming has caused widespread soil micronutrient depletion, leading to an increasing number of people lacking iron, zinc, and vitamin A—micronutrients essential for health and growth (10). This “hidden hunger” impedes progress in child development and maternal health, reinforcing intergenerational cycles of disadvantage and leading to a deterioration in growth (2).

The connection between soil micronutrient depletion (the “Gene” end) and human health is an essential part of the Triple Burden but is often ignored (10). This degradation, intensified by climate change and intensive agriculture, reduces the bioavailability of essential micronutrients in staple crops, directly fueling Hidden Hunger (9). Numerous studies and meta-analyses demonstrate that intensive tillage, high-input monoculture, and the depletion of soil organic matter have resulted in significant reductions in mineral concentrations, including iron, zinc, and magnesium, in staple crops over recent decades, thereby associating degraded soils with diminished nutrient density in the human diet (13). Experimental and field studies further demonstrate that cereals grown on micronutrient-deficient or degraded soils contain significantly less bioavailable iron and zinc, and that long-term nutrient depletion and soil structure degradation are major drivers of micronutrient-poor harvests in many regions. Additionally, prolonged nutrient depletion and soil structure degradation are significant contributors to micronutrient-poor harvests in numerous regions (14).

This basic failure exists alongside and may exacerbate stunting and adult obesity (2). The framework must integrate a strong equity lens to break the intergenerational cycle of malnutrition. The adverse effects of maternal micronutrient deficiencies—intensified by the ‘Gene’ end failure (soil depletion) and ‘Plate’-level nutritional shortfalls shaped by food environments and social determinants of health—are passed directly to the next generation, leading to stunting and reduced cognitive potential (15). Instead of saying that “poor food choices” are the only cause of malnutrition at the Plate end, the framework sees diets as the result of limited food environments and social factors that affect health. These factors include availability, cost, marketing, time, gender roles, and cultural norms that shape what households can realistically afford to eat (16, 17). Policy interventions at the distribution link (e.g., targeted food subsidies, school feeding) must prioritize pregnant women and infants during the first 1,000 days to ensure the benefits of SFS reforms reach those most vulnerable (18).

Tackling malnutrition in all its forms demands a transformation of the food system, moving from sectoral solutions toward integrated and sustainable approaches that ensure diverse, nutrient-rich foods are available year-round and accessible to all (19). Central to this transformation are innovations in climate-smart agriculture, crop biofortification, and the revalorization of local food biodiversity—all demonstrated in recent empirical studies (20). At the policy level, intersectoral cooperation—bringing together health, agriculture, education, and social protection actors raises the prospects for durable progress towards nutrition goals (21).

Community-based interventions and nutrition networks are equally crucial due to the ability to connect local producers, markets, and consumers through activities like community food programs and school feeding, which strengthens the supply of diverse, nutrient-dense foods while also shaping demand via nutrition education and social norms, clearly supporting supply- and demand-side strategies for change (22). Case studies in countries such as Peru, Indonesia, and South Africa demonstrate that local food development, improved dietary diversity, and reduced dependence on imports yield sustainable, population-wide improvements in nutrition outcomes (23).

**Figure 1:**
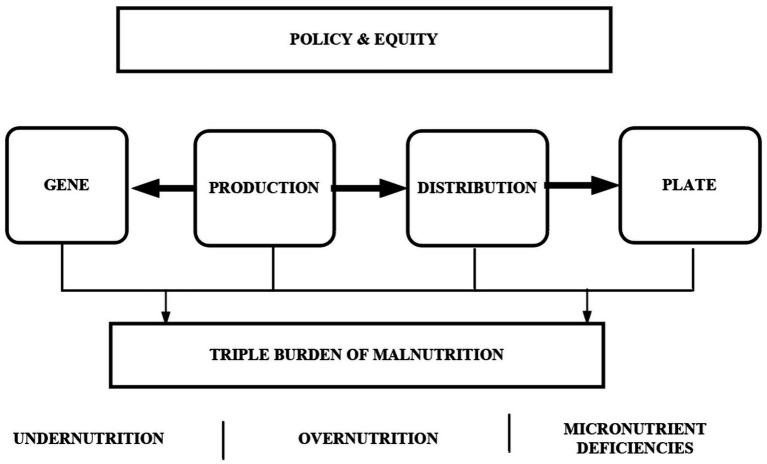
Gene to plate framework and the contribution to malnutrition.

Despite progress, widespread barriers, such as limited political will, societal stigma, and under-resourced institutional networks, frequently undermine large-scale and sustainable gains (24). Not only is it essential to build adaptive, resilient, and sustainable food systems to reach the Sustainable Development Goals, especially the goal of zero hunger, but it is also vital to make communities more resilient to environmental, economic, and health shocks (25). Integrating production, distribution, and nutrition education “from gene to plate” offers a promising pathway to achieve lasting reductions in all forms of malnutrition. The Gene-to-Plate framework also provides a helpful way to address long-standing problems that prevent action on nutrition across sectors. The framework creates a common policy language and a transparent chain of responsibility across several fields, including agriculture, health, education, and social protection by clearly mapping the continuum from soil health and crop genetics (the “Gene” end) to production and distribution, to dietary intake, and to health outcomes (the “Plate” end) (26). This system illustrates how institutional coordination, political will, fragmented financing, and social stigma interact to perpetuate malnutrition, and it clarifies who is responsible for each link, as described in Figure 1 (27). The framework can help with more decisive leadership, long-term commitment, and community-driven design by turning a broad public health issue into a set of specific, connected tasks and by creating process-oriented indicators that can be tracked over time to help policymakers learn and scale up. These indicators include the use of biofortified crops, the nutrient density of basic foods and how budgets are split among different sectors.

## Complex burden: triple forms of malnutrition

Malnutrition is a significant problem worldwide. It’s not just about not getting enough food; it’s also about more people being overweight or obese and not getting enough of essential micronutrients. This triple burden shows how quickly the world is changing (2). These burdens frequently overlap, with households and individuals experiencing stunting, obesity, and hidden hunger simultaneously (3). The undernutrition that stunts growth and impairs cognition in children remains prevalent in many Indonesian districts (28). At the same time, in the same communities, adults face soaring rates of obesity and diabetes, thanks to diet transitions and urbanization (8).

Recent multilevel analyses from an Indonesian study on malnutrition indicate that a significant proportion of children continue to experience stunting, exceeding national averages across multiple regions (29). At the same time, the prevalence of overweight and obesity among adults has risen into the range of 20–30%, illustrating how linear economic growth has not translated into universally improved diets (30). At the same time, survey data continue to document high rates of anemia and other nutrient deficiencies among women and children, suggesting there is a lack in energy requirements and essential micronutrient requirements (31).

This complex pattern appears at the household level. For example, research from rural and peri-urban Indonesia shows mother–child pairs where the parent is overweight or obese and the children are undernourished, or where adults are consuming excessive calories, which might lead to deficiencies in other nutrients (32). Such “double- or triple-burden households” are not rare anomalies but a recurring feature in national and subnational datasets, reflecting shared exposure to food environments dominated by refined staples, sugar-sweetened beverages, and ultra-processed snacks, alongside limited and often unaffordable access to nutrient-dense animal-source foods, fruits, and vegetables (33, 34). The Indonesian Food and Drug Authority (BPOM) rules now focus on back-of-pack nutrient panels and on specific claims such as “no added sugars.” They do not require clear sugar warnings on the front of the package for all high-sugar products (35). Although draft regulations and new policies now propose a Nutri-Label or traffic-light-style front-of-pack label that would grade products based on their sugar, salt, and fat levels, implementation is planned in stages, and full mandatory enforcement is not expected before 2027. National and local studies also show that people living in cities and those with more money tend to eat more energy-dense, nutrient-poor foods (24). On the other hand, structural barriers such as high prices and limited seasonal availability of various food items, exacerbated by inadequate infrastructure, rendered access to a diverse array of healthy meals unattainable for low-income families (36). In Peru, school feeding and community nutrition programs that promoted the use of native Andean crops such as quinoa, amaranth, and biofortified potatoes have been linked to better dietary diversity scores and reductions in stunting in targeted highland regions over the past decade, while also strengthening local value chains for smallholder farmers (37). These actions also confirmed that buying food from local farmers rather than importing it would improve food systems, making them more stable and accessible in the area (38).

In Indonesia, community-based programs that use local staples and home-grown school feeding have been shown to improve children’s minimum dietary diversity and reduce the number of underweight and stunted children, especially in areas with strong nutrition education and women’s empowerment programs (39). At the same time, local food business models and digital platforms that connect rural producers with urban consumers are making it easier for people to buy indigenous foods (40). If these businesses grow and are supported by clear policy, they can gradually reduce people’s reliance on imported wheat-based foods and ultra-processed foods (41).

South African experiences similarly demonstrate the potential of local food systems to address malnutrition while reducing dependence on imports (42). School nutrition programs and community gardens that source vegetables and legumes from smallholders and community producers have improved dietary diversity among school-age children and, in some provinces, contributed to reductions in undernutrition indicators, even in settings facing a high prevalence of overweight and obesity (43). These programs, along with policies that support small farmers and informal markets, show how to produce more food locally and shorten supply chains, thereby easing access to healthy meals, protecting against price volatility, and reducing reliance on imported food (44).

From a sustainable food systems perspective, these data indicate that undernutrition, obesity, and hidden hunger are not separate problems but manifestations of the same underlying weaknesses in how food is produced, processed, distributed, and consumed (45). International reports on sustainable food systems for food security and nutrition emphasize that current systems often prioritize the volume of cheap calories over nutrient quality, rely on input-intensive agricultural practices that degrade soils and reduce the micronutrient content of staple crops, and allow powerful commercial incentives to drive the aggressive marketing of ultra-processed foods (46). They also use farming methods that impair the soil and lower the micronutrient content of staple foods (47). These methods also make it easier for businesses to aggressively market ultra-processed foods. Sustainable food systems, on the other hand, are those that improve food security and nutrition for everyone without hurting the economic, social, or environmental foundations that will allow future generations to have food security and nutrition, which implies protecting soil health and biodiversity, supporting diverse and nutrient-rich production, and ensuring equitable physical and economic access to healthy diets (38). Revealing the complexities of the triple burden, therefore, means tracing these overlapping forms of malnutrition back to shared food system failures and positioning sustainable, nutrition-sensitive food systems as a central strategy for reducing all three simultaneously, rather than addressing each in isolation (48).

## Undernutrition: impact and causes

Insufficient calorie and protein intake during childhood has impacts that extend into adulthood, including impaired intellectual development and lowered economic productivity (28). Insufficient calorie and protein intake in early life is not only a biological problem; it is also a social and economic fault line that shapes how nations such as Peru, Indonesia, and South Africa will develop over the coming decades (37).

Chronic and acute forms—stunting and wasting—illustrate the silent crisis precisely because their long-term costs are largely invisible in daily life yet profoundly visible in national statistics on learning outcomes, labor productivity, and health (49). That still besets millions despite the global increase in agricultural production (50). Poor sanitation, infectious diseases, poverty, and limited access to a diverse range of foods make undernutrition worse for the most vulnerable people. When children do not receive sufficient energy and high-quality protein, which is often the case when they do not get enough of certain micronutrients, the effects go far beyond slow growth. They can also lead to lower school performance, lower earning potential, and a hard-to-eliminate cycle of poverty (51–54).

In Peru, sustained economic growth and targeted social programs have led to an impressive decline in national stunting rates over the last two decades (55). Yet pockets of severe undernutrition persist, particularly in the Andean and Amazonian regions, where poverty, geographic isolation, and limited dietary diversity converge (56). Children in remote highland communities may grow up on monotonous diets centered on starchy staples such as potatoes or maize, with insufficient animal-source foods and limited access to fruits and vegetables, especially outside the harvest season. In these contexts, inadequate intake of both energy and protein is compounded by repeated infections, poor water and sanitation infrastructure, and barriers to health services, resulting in stunting levels that remain unacceptably high despite national gains (57). Several Peruvian studies show that stunted children score lower on cognitive tests and complete fewer years of schooling on average, indicating that nutritional deficiencies in early childhood can adversely affect the quality of human capital and reduce economic productivity in adulthood (44). In the Peruvian Andes, community-based surveys among peasant families report stunting prevalences of about 40% and anaemia rates near 63% in young children, in settings where diets are dominated by potatoes and maize and dietary diversity is low, with protective foods (fruits, vegetables, animal-source foods) consumed on average only one to two times per day (58). Peru’s response has increasingly acknowledged that mere food quantity is inadequate; initiatives such as school feeding programs, community-based interventions, and conditional cash transfer programs are being restructured to emphasize diverse, locally sourced foods, including native Andean grains and legumes, to enhance both the quality and sustainability of children’s diets (59). Where these interventions have successfully integrated nutrition education, water and sanitation improvements, and careful targeting of vulnerable communities, reductions in stunting and underweight have been observed, supporting the argument that undernutrition is deeply embedded in broader food-systems and social conditions rather than simply in household food choices (37). Yet the persistence of high stunting rates in certain regions reminds policymakers that overcoming the legacy of insufficient protein and calorie intake requires long-term investments in infrastructure, equitable access to services, and the empowerment of rural and indigenous people, whose food systems have traditionally been overlooked (38).

Indonesia is another clear example of how undernutrition can persist in a middle-income country despite increasing food availability and accessibility (60). National surveys consistently report stunting prevalence above global targets, with some provinces and districts experiencing rates above 30%, particularly in poorer and more remote areas (39). Although total caloric intake has increased, many children still consume diets dominated by polished rice (61). While total calorie intake has increased, many children still consume diets dominated by polished rice and other refined staples, with limited intake of high-quality protein sources such as eggs, fish, or meat, and inadequate consumption of fruits and vegetables (61). This reliance on low-cost, low-diversity diets reflects structural factors—income constraints, food prices, market access, and cultural norms—as well as the aggressive expansion of ultra-processed products that displace traditional, nutrient-dense foods (31).

In some rural areas, poor sanitation, unsafe drinking water, and high rates of microbial infections lead to unhealthy eating (62). This causes environmental enteric dysfunction and impairs the body’s ability to absorb nutrients. Multilevel analyses demonstrate that children enduring multiple infections and residing in households with insufficient sanitation are considerably more vulnerable to undernourishment, even when controlling for household wealth and maternal education (31). These children are more likely to start school with lower cognitive readiness, and cohort studies show that stunted Indonesian children get less education and make less money as adults (63). This shows a direct link between insufficient early intake of calories and protein and a decline in national productivity. The economic implications are not abstract; modelling studies at the country and regional levels estimate that the cumulative cost of child undernutrition—including lost earnings, higher health expenditures, and reduced economic growth—amounts to several percentage points of GDP each year in high-burden countries (15).

At the same time, Indonesia is experiencing rapid urbanization and dietary change, resulting in the coexistence of undernutrition and overnutrition within the same communities and even within households (24). In peri-urban settlements, children may still be stunted or underweight because their diets are low in overall quality and quantity. At the same time, adults increasingly consume sugar-sweetened beverages and ultra-processed snacks (64). This dual exposure demonstrates that insufficient calorie and protein intake in early life is not simply a reflection of food scarcity; it also shows that the food system is unable to provide the most at-risk people with many affordable, nutrient-dense choices, even though cheap, energy-dense foods are available in the market (65).

In South Africa, the long-term effects of childhood malnutrition vary but remain equally strong. Despite social protection programs and better access to food, national and provincial surveys show that a large number of South African children are still stunted or underweight. This is especially true in poor rural provinces and informal urban settlements. Many of these children grow up in places where they consume a lot of processed, high-calorie foods, such as refined maize meal, bread, and other energy-dense foods. They do not have easy access to animal protein, fresh fruits, and vegetables due to cost, availability, and market structure (41, 44). Evidence from South Africa similarly illustrates the capacity of local food systems to combat malnutrition while diminishing reliance on imports (66, 67). Research from South Africa has repeatedly demonstrated that stunted children have poorer school readiness. They perform worse on tests of memory, attention, and language, and they are more likely to drop out of school early, which limits their future job prospects and earnings. These individual problems add up to a macroeconomic problem: fewer skilled workers, lower productivity, and higher health care costs, driven by both the long-term effects of insufficient food and the rise in diet-related chronic diseases (49). Moreover, the South African food environment—characterized by wide availability of cheap processed foods in both urban and rural areas—means that children who have experienced early undernutrition may later become overweight or obese as their diets change, which raises the risk of metabolic disease and makes the economic and social costs even higher (44, 68).

Across these three countries, the pattern is clear: chronic and acute undernutrition are not just vestiges of the past but active drivers of present and future inequality. Poor sanitation and infectious disease create a vicious cycle in which inadequate calorie and protein intake is further undermined by repeated illness and reduced nutrient absorption (69). At the same time, poverty and limited access to diverse, high-quality foods constrain children’s diets, preventing them from supporting optimal growth and development (70). At the same time, macro-level changes in food systems—such as the dominance of refined staples, concentration of food retail, and the spread of ultra-processed products—expose the most vulnerable children to an environment that makes healthy eating both difficult and costly (55, 71, 72).

A compelling interpretation of the experiences from Peru, Indonesia, and South Africa indicates that undernutrition is neither unavoidable nor solely a matter of personal choice. Policies that have intentionally strengthened local food systems—by helping small farmers, diversifying production, improving diets in schools and communities, and investing in water, sanitation, and health services—have led to lower rates of stunting and wasting (15, 69). On the other hand, when these investments have been inconsistent, short-lived, or not tied to broader changes in the food system, undernutrition has remained stubbornly high (49). This means the leading causes are poverty, as shown by structural inequalities in land access, services, markets, and power. Recognizing the lasting cognitive and economic effects of insufficient calorie and protein intake in early childhood underscores the imperative for governments and stakeholders in Peru, Indonesia, and South Africa to prioritize sustainable, nutrition-sensitive food systems and integrated social policies (31, 41, 44, 68).

Table 1 summarizes the analysis criteria and key indicators for nutrition strategies and food systems in Peru, Indonesia, and South Africa, with a focus on empowering local sources.

**Table 1:** Comparative analysis of multisectoral nutrition strategies and local food development.

Analysis criteria	Key indicators	Peru	Indonesia	South Africa
Community-based program	Program focus	Focus on the utilization of local agrobiodiversity and conditional cash transfers.	Convergence of stunting programs at the Integrated Health Post (*Posyandu*) level; education on local carbohydrate diversification	Urban/peri-urban community garden initiatives and support for local agrifood small businesses
Multisectoral integration	Coordination mechanism	Strong political commitment, transparent budget allocation, and centralized indicators	Coordination through task forces and budget convergence from various ministries/agencies	Challenges in harmonizing agrarian, health, and trade policies due to historical inequality issues
Local food and dietary diversity	Import substitution strategy	Revitalization of Andean root and tubers (quinoa, amaranth, etc.) and promotion of consumption	Utilization of sago, cassava, and sorghum as alternatives to wheat for import substitution	Support for emerging farmers and integration of local agricultural products into formal supply chains
Key sustainability outcomes	Reducing malnutrition and import dependence	Significant reduction in stunting (numbers), but processed food imports are still high.	Stunting has declined gradually, but dependence on wheat imports remains a challenge.	High disparity in malnutrition between regions, high food availability, but low food access

Peru, Indonesia, and South Africa applied a community-based, multisectoral programmatic approach to address malnutrition through local agrobiodiversity and social protection programs (73). Peru shows strong political will and local crop revitalization; Indonesia focuses on stunting reduction program and food source diversification; South Africa promotes community gardens and small agrifood enterprises. All face persistent import dependence, ultra-processed food issues, socioeconomic inequalities and inequities in dietary access, and malnutrition outcomes.

## Rising overnutrition and diet-related disease

Modern urban life, expanding income, and an explosion of convenience foods have brought a new enemy: excess calories with insufficient essential micronutrients (74). Obesity, hypertension, and type 2 diabetes are increasing in low- and middle-income countries (75–77). This “double-triple burden” required a firm policy that challenged the old intervention model (50).

In Peru, Indonesia, and South Africa, high consumption of ultra-processed foods has deteriorated dietary habits, leading to increases in non-communicable diseases and overnutrition. At the same time, undernutrition remains a public health problem. As incomes grow and people move to cities, diets shift away from traditional staples and minimally processed foods toward packaged snacks, sugary drinks, and fast food, delivering excess calories but few essential nutrients. This nutrition transition is not a simple story of “too much food”, but of the wrong kinds of food being affordable, ubiquitous, and massively advertised, especially to poor families that still struggle with food security (31, 44).

Peru’s experience shows how this happens when progress brings new risks. National health surveys show that the country has seen significant economic growth and a sharp decline in the number of malnourished children, while overnutrition is becoming more common, along with a growing burden of non-communicable disease, especially in urban and peri-urban areas (59). There are now many supermarkets, convenience stores, and fast-food restaurants in Lima and other cities. These modern outlets sell cheap, tasty, high-calorie foods, whereas in low-income areas, access to fruits, vegetables, and animal-based foods is more challenging. In the meantime, advertising for sugar-sweetened beverages and ultra-processed snacks targets children and adolescents, creating new consumption norms that diverge sharply from traditional dietary patterns based on native grains, tubers, and legumes (31, 44, 68).

Within this changing food environment, these patterns show that overnutrition and undernutrition are not separate problems, but rather the result of the same structural forces acting in different ways: poverty, unequal access to healthy foods, and a market system that favors the sale of ultra-processed foods. In response, Peru has started placing warning labels on the front of food packages that are high in sugar, salt, and fat (78). It has strengthened school food policies, showing that the government recognizes the rise in diet-related diseases as a systemic problem that cannot be solved by providing health-related information alone. As the food industry evolves rapidly, policymakers must enact rules that adapt to a dynamic landscape that continually introduces new ideas and products (59).

Indonesia illustrates a similar dynamic, and in some respects even more complex, in the coexistence of stunting, overnutrition, and non-communicable disease among adults (65). National and provincial data reveal that in many communities, thin or stunted children share a household with parents who are overweight or obese, reflecting diets that are simultaneously inadequate in quality for children and excessive in energy for adults (32). The rapid expansion of modern retail and online food platforms has increased the availability and accessibility of energy-dense foods. These low-fiber foods are often cheaper and more frequently promoted than nutrient-dense foods (44). This pattern corresponds with global characterizations of the nutritional transition in low- and middle-income nations (32). For adults who may have experienced undernutrition in their childhood, this change can lead to a pattern in which early growth problems are followed by rapid weight gain later in life, which raises the risk of diabetes and high blood pressure (44). Research conducted in Indonesia and the surrounding region demonstrates significant correlations between consumption of ultra-processed foods and elevated body mass index, waist circumference, and metabolic risk factors, consistent with findings from other global contexts (30). Having stunted children and overweight adults living in the same house is not unusual; it is a natural result of a food system that does not give children enough healthy food and offers adults a lot of cheap, high-calorie foods (79).

Policy debates in Indonesia are increasingly recognizing that voluntary measures and nutrition information on the back of packages are not enough to stop the rise of overnutrition and diet-related diseases. Draft rules requiring labelling sugar, salt, and fat on the front of packages, as well as ideas to make it harder to market unhealthy foods to kids, show that the government is moving toward more structural changes to the food environment rather than just asking people to change their behavior. But there are still problems with implementation timelines, industry pushbacks, and enforcement capacity. This shows the “puzzle” of the policy of protecting public health in a situation where economic interests and consumer preferences are strongly aligned with the growth of convenience foods (35).

The path South Africa is on shows even more how rising overnutrition and diet-related diseases can take over a country while undernutrition remains the same. The government now has a high prevalence of overweight and obesity in sub-Saharan Africa, especially among women. It also has a lot of people with hypertension, type 2 diabetes, and heart disease. At the same time, child stunting and being underweight are still common in poor rural provinces and informal urban settlements. This creates a striking “double burden” at the national, community, and household levels. A small number of stores and a lot of cheap, ultra-processed foods make up South Africa’s food environment. In many urban and rural areas, fast-food places, corner stores, and informal vendors mainly sell refined starches, fried snacks, and sugary drinks, making it hard to find affordable, fresh products (44, 68).

Research shows that many South African adults, especially those with low incomes, consume excessive amounts of refined carbohydrates, fats, and added sugars, but not enough fiber or micronutrients. This pattern is linked to the rapid rise in obesity and metabolic disease. Kids who grow up in these kinds of places may not grow as tall because they are lacking in nutrition and frequently have diseases. However, when they consume high-calorie diets, they may gain weight excessively, which puts them at a higher risk for chronic diseases in the future. The coexistence of undernutrition and overnutrition within the same families and communities thus reflects deep structural inequities in access to healthy food, influenced by historical patterns of land dispossession, urban planning, and economic policy. South Africa’s policy response, including a tax on sugar-sweetened beverages and efforts to improve school nutrition, represents necessary steps toward reshaping the food environment. These steps, however, need to be part of a larger plan to improve fruit and vegetable availability in the area, change how food is sold, and address the social factors that affect diet (44, 68).

The experiences of Peru, Indonesia, and South Africa demonstrate that the high incidence of overnutrition and non-communicable diseases does not indicate a resolution of malnutrition; instead, it signifies a transition in food systems that fails to address the problem. In all three countries, modern urban life, rising incomes, and the expansion of convenience foods have created environments in which excess calories and poor nutrient quality coexist with persistent child under nutrition. The “double burden” of stunted children and overweight adults sharing the same home is therefore a powerful indicator that traditional intervention models—focused either on food supplementation for the poor or on lifestyle advice for the affluent—are no longer adequate. Instead, these patterns call for a new generation of policies that approach malnutrition as a systemic outcome of how food is produced, priced, marketed, and regulated, and that work simultaneously to protect children from undernutrition and adults from unhealthy diets and chronic disease (31, 38, 44).

## Hidden hunger: micronutrient deficiencies

Micronutrient deficiencies, such as vitamin A, iron, zinc, and iodine, quietly sabotage the health, immunity, and development of billions of people worldwide (80). Soil nutrient depletion from intensive agriculture and climate change exacerbate the problem, reducing the nutritional value of staple crops, even as caloric supply increases (81). Additionally, prolonged nutrient depletion and soil structure degradation are significant contributors to micronutrient-poor harvests in numerous regions (14). Hidden hunger presents in children as weakened immunity, delayed milestones, and an increased risk of infectious and chronic diseases (82).

In Peru, Indonesia, and South Africa, hidden hunger is the invisible face of malnutrition that persists even when plates appear complete, and calorie supply has increased. Iron, vitamin A, zinc, and iodine deficiencies remain widespread among women and children, undermining immune function, cognitive development, pregnancy outcomes, and long-term productivity. What makes this form of malnutrition particularly insidious is that it does not usually make people look skinny or short; instead, it slowly lowers the quality of life by making people frequently sick, causing cognitive problems, and raising the risk of death for vulnerable groups. (31, 44).

In Peru, national surveys have documented substantial burdens of anemia in young children and women of reproductive age, especially in rural Andean and Amazonian regions where diets are monotonous and dominated by starchy staples. Children may receive enough calories from potatoes, rice, or maize. Still, a limited intake of animal-source foods, fruits, and vegetables means that iron, zinc, and vitamin A requirements are not met. In some mountainous regions, intensive farming and soil erosion cause micronutrient depletion, resulting in less nutritious staple crops, even when yields are excellent. In these communities, hidden hunger leads to more respiratory and gastrointestinal infections, worse cognitive outcomes, and lower school performance, which keeps many kids stuck in cycles of disadvantage that last into adulthood. Peru’s efforts to address these problems, such as adding iron to foods, enriching them with nutrients, and promoting native crops, show that fighting hidden hunger requires coordinated action across agriculture, health, and education, not just medical treatment (38, 44, 68, 73).

Indonesia faces a similarly complex challenge. Despite improvements in food availability, anemia and other micronutrient deficiencies remain highly prevalent among pregnant women and young children, particularly in poorer and remote provinces. Diets centered on polished rice, with limited intake of animal-source foods and dark-green leafy vegetables, provide energy but insufficient iron, zinc, and vitamin A. Studies also point to low dietary diversity and suboptimal feeding practices during the first 1,000 days of life, a critical window when micronutrient deficits can cause irreversible damage to growth and brain development. Environmental factors, such as degraded soils and climate-related shocks that impact local food production, can diminish the micronutrient density of staples and decrease the availability and affordability of nutrient-rich foods. The results are precise: high rates of infectious disease, poor school performance, and the passing on of poor nutrition from one generation to the next. This shows that hidden hunger is both a health and a development emergency (44, 68).

In South Africa, hidden hunger coexists with high levels of overweight, obesity, and diet-related non-communicable diseases, demonstrating that micronutrient deficiency is not confined to food-insecure households alone. National and provincial assessments report a significant prevalence of anemia and vitamin A deficiency among children and women, even in communities where calorie intake is sufficient or excessive. Diets dominated by refined maize meal, white bread, and cheap processed foods often lack the micronutrient density needed for healthy growth and immunity, especially when intake of fruits, vegetables, and animal-source foods is constrained by price, availability, and retail structure. Although South Africa has implemented large-scale food fortification policies, including the enrichment of staple flours with iron and other nutrients, gaps remain in coverage and quality, particularly regarding dietary diversification and infection control (44, 68).

The same story is true in Peru, Indonesia, and South Africa: hidden hunger thrives where food systems prioritize volume and low cost over nutrient density, and where soil health, crop diversity, and fair access to micronutrient-rich foods are not well cared for. Kids may no longer look sick, but they still have the biological scars of not getting enough vitamins and minerals. This weakens their immune systems, lowers their cognitive potential, and makes it harder for them to learn and earn money over their whole lives. To fight hidden hunger in these places, we need more than just campaigns to get people to eat more; we need to change the way food is grown and distributed. This means literally restoring soil fertility and promoting biofortified, diverse crops, and socially ensuring that nutrient-dense foods are affordable, desirable, and available to those who need them (38).

## From fragmented fixes to systemic solutions, genetic innovation and biodiversity, and climate-smart and regenerative agriculture

Peru, Indonesia, and South Africa clearly show why short-term feeding projects, vitamin capsules, or isolated behavior-change campaigns should work together to break the cycle of undernutrition, hidden hunger, and rising diet-related non-communicable diseases. Each country has made several changes to health, agriculture, and social protection and has also implemented multiple “fixes” in those fields. Yet malnutrition in all its forms persists precisely because the underlying food systems still prioritize yield, uniformity, and cheap calories over diversity, nutrient density, and ecological resilience. Moving from fragmented fixes to systemic solutions requires re-engineering what is grown, how it is made and how it gets to people’s plates. Genetic innovation, biodiversity, and climate-smart, regenerative agriculture should be at the center of policy and practice, not on the outside (38, 44, 68).

Peru is an excellent example of how genetic innovation and agrobiodiversity can significantly impact a food system. Peru is the origin home for roots and tubers. These can be applied to solve both malnutrition and climate change. Breeding programs and biofortification initiatives have developed iron-rich beans, zinc-enhanced potatoes, and high-protein or high-micronutrient quinoa varieties. At the same time, public policies increasingly encourage their use in school feeding and social protection schemes. At the same time, climate-innovative and regenerative practices—such as diversified rotations, conservation agriculture on steep Andean slopes, and the revival of traditional water-harvesting and soil-management techniques—are helping smallholders adapt to shifting rainfall patterns and temperature extremes. When these agronomic innovations are linked to nutrition-sensitive procurement and market development, the result is not just a more resilient farming system, but a food system that can consistently deliver micronutrient-dense local foods to vulnerable children and communities (44).

Indonesia’s situation underlines both the costs of neglecting diversity and the potential of reclaiming it. Decades of policies that privileged rice self-sufficiency have narrowed production and diets, often at the expense of traditional staples such as sorghum, sago, local tubers, and a wide variety of leafy vegetables and legumes. This monoculture bias has contributed to hidden hunger and left smallholders exposed to pest outbreaks, soil degradation, and climate shocks. In response, researchers and local governments are beginning to invest in improved and biofortified varieties of underutilized crops, including nutrient-dense local tubers and pulses, alongside efforts to reintroduce them into school feeding, village nutrition programs, and community enterprises. Parallel initiatives in climate-smart and regenerative agriculture—such as integrated rice–fish systems, agroforestry with fruit and timber species, and organic or reduced-input practices—aim to restore soil health, enhance biodiversity, and stabilize yields amid increasingly erratic climate conditions. When Indonesia consciously connects these “Gene” and “Field” innovations with nutrition-focused policies and markets, the country can move beyond short-term supplementation toward a healthier, more varied, and more sustainable food system (31, 44, 79).

South Africa, meanwhile, stands at a critical junction between a highly industrialized food system and the need to re-embed biodiversity and ecological sustainability. Diets dominated by refined maize, wheat, and ultra-processed foods have fueled both micronutrient deficiencies and soaring obesity and non-communicable diseases. Sorghum, millet, cowpeas, and Bambara groundnuts are among the native grains and legumes grown in South Africa. There are also leafy vegetables that can grow in dry conditions and do not require many nutrients. Some projects in schools and communities are working with smallholder farmers who use organic soil-building methods, conservation agriculture, and minimum tillage to grow these crops. Research programs are exploring ways to improve and make these crops more nutritious. Some of the native grains and legumes grown in South Africa include sorghum, millet, cowpeas, and Bambara groundnuts. There are also leafy greens that can grow in dry conditions and do not require many nutrients. Some school and community projects are working with small farmers who use organic methods to build soil health, such as conservation agriculture and minimum tillage. Research programs are seeking ways to improve these crops and make them more nutritious. People are promoting cover crops, agroforestry, and holistic grazing as climate-smart and regenerative ways to rebuild soil organic matter and reduce the likelihood of drought and extreme heat in water-scarce areas. When combined with public procurement policies that prioritize locally produced, nutrient-rich foods for schools and clinics, these initiatives can gradually shift the food system away from imported or heavily processed staples and toward a diversified, climate-resilient base that supports both human and ecosystem health (44, 68).

**Figure 2:**
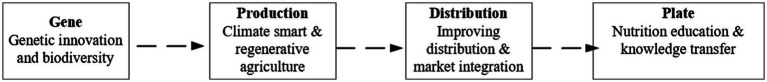
The involvement of the gene-to-plate chain in the food system.

The lesson learned in Peru, Indonesia, and South Africa is that genetic innovation, biodiversity, and climate-smart, regenerative agriculture are not just extra things to add on; they are the basis of any real systemic solution to malnutrition. Breeding crops that are more nutritious and can survive in different climates, restoring soils that are diverse and alive, and redesigning landscapes to buffer climate shocks are powerful nutrition interventions when linked to inclusive markets, nutrition-sensitive procurement, and social policies that ensure the resulting foods reach those most at risk. Only by knitting these elements together—“from gene to field to plate”—can countries move beyond fragmented fixes and build food systems that are capable of sustaining healthy, diverse diets in an era of ecological and economic uncertainty (41, 44, 68). Figure 2 describes how the involvement of the gene-to-plate in the food system.

## Improving distribution and market integration, local innovation and successes

In Peru, Indonesia, and South Africa, the way food moves—from smallholder fields to village markets and urban plates—often decides if nutritious crops really lead to better diets. In Peru, efforts to include Andean smallholders in school feeding and social protection programs have shown that public procurement can improve children’s access to a broader range of nutrient-rich foods and boost local markets for native grains, legumes, and vegetables. When municipalities hire local producer groups instead of wholesalers from far away, they shorten supply chains, reduce reliance on imported staples, and create stable demand that encourages farmers to invest in a wide range of high-quality production (38, 44, 68).

Some of Indonesia’s local innovations are digital platforms and community businesses that link small farmers of tubers, vegetables, and fish directly with city dwellers. This cuts out some middlemen and makes it easier for prices to move. These models, along with school feeding programs and village-level nutrition programs, have made it easier for people in participating areas to access fresh, local foods and reduced their risk of global price shocks. Connecting smallholder and community producers with school nutrition programs and informal urban markets in South Africa has increased the availability of vegetables, legumes, and native crops in low-income neighborhoods. This shows that distribution and market design are just as important as production innovations for making food systems healthier and fairer (83).

## Nutrition education and knowledge transfer, coordinated policy and multisectoral networks

In Peru, Indonesia, and South Africa, nutrition education and coordinated, multisectoral action are becoming more critical to ensuring that food system changes actually lead to better health. School feeding in Peru has changed from simply providing kids with calories to a place where kids, teachers, farmers, and local officials learn together about healthy eating, the value of fresh, minimally processed foods, and the importance of regional biodiversity. Programs that include food and nutrition education in the curriculum, along with school gardens and buying food from local farmers, have shown that kids not only eat better at school but also bring new tastes and knowledge home, which encourages community members to seek more healthy, varied foods. These efforts work best when the authorities responsible for education, health, agriculture, and social development plan and budget together. This makes school meals a real multisectoral policy tool instead of just a stand-alone program (84).

Indonesia’s efforts to reduce stunting show how complex and important it is to build nutrition networks that span many sectors. The National Strategy to Accelerate Stunting Prevention calls for “convergent” interventions that integrate health services, water and sanitation, social protection, agriculture, and intensive caregiver nutrition education. These interventions should be available from the village level to the national level. Policy analyses indicate that improvements in nutritional literacy, counselling for pregnant women and parents of young children, and community empowerment can substantially enhance the efficacy of cash transfers and food-based interventions, contingent on the coordination of targets, data, and financing by ministries and local governments. Strengthening governance—through more explicit mandates, joint planning, and shared monitoring systems—is therefore as important as teaching families about balanced diets, because without coordinated policy, even good educational messages are undermined by food environments dominated by cheap, ultra-processed products (63, 85).

The National School Nutrition Program (NSNP) in South Africa demonstrates how nutrition education and cross-sectoral networks can be made permanent at scale. The NSNP not only provides millions of students in the poorest schools with meals every day but also teaches them about nutrition and encourages them to grow their own food in school gardens. The program is based on cooperation between the education, health, agriculture, and social development sectors. It is supported by a national food and nutrition security plan that views school feeding as part of a broader effort to combat hunger and malnutrition. When these networks work well, schools become places where kids, parents, and small-scale producers can learn and strengthen the local food system. This changes how people eat over time, making them more resilient to food insecurity (86).

## Overcoming barriers and inspiring progress

The most significant barriers—weak institutional coordination and political inertia—can be mitigated by the gene-to-plate framework. By providing a clear, evidence-based diagram, the framework creates a shared policy language and a transparent accountability chain. It transforms a vague public health problem into a set of actionable, interlinked policy mandates, thereby encouraging sound leadership and long-term commitment prerequisite to scaling up wins (56, 87). Future research derived from the Gene-to-Plate framework ought to employ systems not only to assess health outcomes but also to assess progress across all links. Success indicators should include the variable at which people start eating biofortified crops (Gene), the nutrient density of the staples people consume (Distribution/Plate), and the levels of multisectoral budget allocation (Policy). This focus on process metrics, rather than just final prevalence rates, allows policymakers to identify and correct failures at specific points along the continuum. The Gene-to-Plate model is not just a concept; it also creates barriers to persistence. Weak institutional coordination, insufficient funding, political inertia, and social stigma limit the persistence of obstacles that contribute to malnutrition. To make these wins last and drive systemic change, we need strong leadership, a long-term commitment, and a design rooted in the community. The ‘Gene-to-Plate’ framework can help get past the problems of political inertia and poor coordination between institutions by involving the intermediate mechanisms through the market. The movement of food from small-holder fields to markets is a necessary condition, but it is not sufficient to guarantee improved diets without addressing these additional barriers. This framework makes it clear how soil conditions and public health are connected, giving everyone a common language, policy and a clear chain of responsibility. It makes policymakers from both the health and agriculture sectors work together. To recognize that action on crop genetics (the ‘Gene’ end) directly supports the reduction of obesity and hidden hunger (the ‘Plate’ end), thus fostering the long-term commitment and sound leadership required for systemic transformation (88).

## Conclusion

By advancing the Gene-to-Plate framework as a way of re-thinking the triple burden of malnutrition, not as three separate problems, but as interconnected outcomes of how food systems function from soil and seed to the plate and beyond. Drawing primarily on Indonesia, with comparative insights from Peru and South Africa, we highlight current challenges, including degraded soils and narrow crop portfolios at the Gene and Production ends, inequitable and import-dependent value chains at the Distribution end, and Plate-level dietary patterns shaped by constrained food environments and social determinants rather than “poor choices” alone. Building on this analysis, this article argues that future work should focus on applying and refining the framework through systems modelling, policy tracking, and empirical evaluations of Gene-to-Plate interventions. By doing so, the Gene-to-Plate perspective can guide governments, practitioners, and researchers toward integrated, context-specific strategies that move beyond fragmented fixes and support sustainable reductions in all forms of malnutrition. From gene to plate, addressing the triple burden of malnutrition requires transforming food systems to ensure that climate-smart production, diversified local value chains, and nutrition education jointly secure year-round access to affordable, nutrient-dense foods for all. Countries such as Indonesia, Peru, and South Africa can break the cycle of undernutrition, hidden hunger, and diet-related diseases that have been passed down from generation to generation. They can do this by implementing multisectoral policies, empowering community-based nutrition networks, and ensuring that equity is integrated across the entire food system, from soil and seed to consumption.

## Data Availability

Publicly available datasets were analyzed in this study. This data can be found at: https://openknowledge.fao.org/items/1516eb79-8b43-400e-b3cb-130fd70853b0.
